# Cord Blood DNA Methylation Biomarkers for Predicting Neurodevelopmental Outcomes

**DOI:** 10.3390/genes7120117

**Published:** 2016-12-03

**Authors:** Nicolette A. Hodyl, Claire T. Roberts, Tina Bianco-Miotto

**Affiliations:** 1 Department of Neonatal Medicine, Women’s and Children’s Hospital, Adelaide 5006, Australia; 2 School of Medicine, University of Adelaide, Adelaide 5005, Australia; claire.roberts@adelaide.edu.au; 3 Robinson Research Institute, University of Adelaide, Adelaide 5005, Australia; tina.bianco@adelaide.edu.au; 4 School of Agriculture, Food and Wine, Waite Research Institute, University of Adelaide, Adelaide 5005, Australia

**Keywords:** epigenetics, DNA methylation, neurodevelopment, cord blood

## Abstract

Adverse environmental exposures in pregnancy can significantly alter the development of the fetus resulting in impaired child neurodevelopment. Such exposures can lead to epigenetic alterations like DNA methylation, which may be a marker of poor cognitive, motor and behavioral outcomes in the infant. Here we review studies that have assessed DNA methylation in cord blood following maternal exposures that may impact neurodevelopment of the child. We also highlight some key studies to illustrate the potential for DNA methylation to successfully identify infants at risk for poor outcomes. While the current evidence is limited, in that observations to date are largely correlational, in time and with larger cohorts analyzed and longer term follow-up completed, we may be able to develop epigenetic biomarkers that not only indicate adverse early life exposures but can also be used to identify individuals likely to be at an increased risk of impaired neurodevelopment even in the absence of detailed information regarding prenatal environment.

## 1. Introduction

### 1.1. Neurodevelopment

Neurodevelopment begins early in fetal life, with neuronal migration, synaptogenesis and myelination of neurons occurring from the second month of pregnancy [1]. Environmental exposures in utero can disrupt these maturational processes, affecting normal postnatal motor, cognitive and behavioral development. In school-aged children, poor neurodevelopment is reflected by reduced cognitive and motor performance, and poor voluntary regulation of attention, emotion and behavior. These factors predict reduced academic and economic success in adulthood, worse health outcomes and reduced life expectancy [2]. Even subtle alterations in brain structural development and connectivity that occur in the perinatal period can have a significant impact on health and socioeconomic outcomes persisting into adulthood [3,4]. This underscores the need to identify early determinants of poor neurodevelopment, so that interventions can be introduced in early childhood, when the brain is most plastic and responsive to treatment [5].

### 1.2. Current Predictors of Neurodevelopmental Outcome

Our current ability to predict poor motor, cognitive and neurobehavioral outcomes in the neonatal period is limited. Neuroimaging has achieved the most attention as a tool for early prediction and monitoring of development. Cranial magnetic resonance imaging (MRI) allows for qualitative assessment of grey and white matter injury and is superior to ultrasound imaging for the detection of white matter changes [6]. While MRI predicts cerebral palsy with sensitivity double that of ultrasound [7], MRI imaging of preterm infants at term corrected age predicts functional and neurobehavioral impairment with only reasonable accuracy. While clinical assessments of neuromotor and neurological function, specifically Prechtl’s General Movements, have been found to have good predictive accuracy for the outcome of cerebral palsy [8], there are limitations in their utility to predict other neurodevelopmental impairment. Neonatal neurological examination is a poor predictive tool with sensitivity for poor neurodevelopmental outcome of 57%–86% and specificity of 45%–83% when performed before term age in preterm infants [9]. Similarly, the Neonatal Intensive Care Network Neurobehavioral Scale (NNNS) and Assessment of Preterm Infants Behavior (APIB) are the most valid measures of neuro-behavior yet have poor reliability and limited clinical utility [8].

Given the current limitations of accurately assessing future neurodevelopmental outcomes, identifying cord blood biomarkers which aid in the prediction of later cognitive, motor and behavioral function would be a significant step towards targeted, effective interventions implemented at a time-point where their effects are likely to be greatest. DNA methylation in cord blood represents one such potential biomarker that is gaining research momentum. This epigenetic process reflects the biological convergence of perinatal exposures that can have important long-term consequences. Here we review literature assessing the potential for epigenetic biomarkers in cord blood to be used to predict poor neurodevelopmental outcome. While most studies to date have employed targeted gene approaches, the capacity for genome wide arrays to identify novel epigenetic biomarkers will also be discussed.

## 2. Epigenetics

Epigenetic modifications alter gene expression without changes to the DNA sequence and are often reversible. Typically epigenetic modifications encompass DNA methylation, histone modifications and non-coding RNAs. DNA methylation is the most commonly studied epigenetic modification and typically involves the addition of a methyl group to a cytosine adjacent to a guanine, denoted CpG, however cytosine methylation also occurs at CH sites (where H is A, C or T) and this has been repeatedly reported in the brain (reviewed in [10]). The mechanisms by which DNA methylation regulate gene expression are complex and have been reviewed elsewhere (see [10,11]). In general, DNA methylation is a reversible modification associated with gene regulation, particularly when present in CpG rich regions associated with gene regulatory regions [12]. However, how DNA methylation regulates gene expression is dependent on the genomic sequence context in which it occurs. One way DNA methylation can alter gene expression is by altering chromatin compaction and in doing so alter accessibility of transcription factors. Histone modifications refer to modifications of lysine and arginine residues in histone tails and include acetylation, methylation, phosphorylation, sumoylation and ubiquitination. Histone modifications regulate gene expression by altering the chromatin. Non-coding RNAs, such as microRNAs or long non-coding RNAs can also regulate gene expression without altering the DNA sequence by binding to mRNA resulting in gene repression or by blocking protein translation.

### 2.1. DNA Methylation

DNA methylation is the most studied epigenetic change. It has a critical role in development [12] and due to its stability and ease of measurement is widely investigated both to understand mechanisms driving disease states, as well as for its potential to be used as a biomarker for numerous health and disease outcomes [13,14,15,16,17,18]. DNA methylation marks have been associated with diseases such as cancer, and therefore show promise for use as independent biomarkers. DNA methylation has also been used as a measure or predictor of environmental exposures [19], with DNA methylation in blood reflecting environmental exposures such as infection, diet, smoking, exposure to toxins and heavy metals (reviewed in [17]). DNA methylation also plays an important role in imprinting, and imprinting disorders have been associated with poorer neurodevelopmental outcomes (see reviews [20,21,22,23]).

### 2.2. Tissue Specificity

One of the many advantages of studying DNA methylation is the ease with which it can be assessed in peripheral bodily fluids such as blood, urine, sperm, stool samples and saliva [19]. The use of epigenetic biomarkers in cancer is widely studied with a large number of reports investigating the use of DNA methylation biomarkers for diagnosis, predicting prognosis and therapeutic response. Numerous studies have repeatedly shown that DNA methylation changes in blood can be used as a biomarker for predicting cancer risk [13,15,19,24].

Early studies that assessed brain DNA methylation patterns and their association with offspring behavior were conducted in rodents where environmental exposures are amenable to manipulation. For example, alterations in glucocorticoid receptor *(Nr3c1)* expression in the brain due to differences in maternal behavior are associated with DNA methylation of *Nr3c1* [25]. That study highlighted how neurodevelopmental behavior can be altered in offspring due to maternal care and that gene expression and corresponding DNA methylation can also be altered. These studies assessed DNA methylation as a mechanism related to regulation of gene expression in that specific tissue and under those conditions. The use of DNA methylation from cord blood as a biomarker for neurodevelopmental outcome raises the issue of tissue specificity. Does measuring DNA methylation changes in peripheral fluids such as cord blood reflect what is happening in the brain? A recent study compared DNA methylation profiles in blood and four different brain regions and found that the whole blood DNA methylation profile was distinctively different from the brain regions [26]. However, when the analysis was performed only with the blood variable probes (185,060 instead of 427,018 probes) then there was evidence of significant correlations between blood and brain DNA methylation profiles at 1.5% of the sites [26]. However, we are in agreement with the authors of that study that it does not matter if cord blood DNA methylation patterns do not reflect the same DNA methylation status of genes in the brain, if they alone are associated with (and potentially predict) a poor outcome. Hence, the use of DNA methylation changes in blood as a biomarker of subsequent neurodevelopmental outcomes can potentially be a very powerful and informative tool in human studies. In such studies, DNA methylation in blood is used as an environmental biomarker that can predict future outcomes. With current levels of understanding however, caution with over-interpreting these findings is warranted, as cord blood contains a mixture of cell types (including monocytes, lymphocytes and granulocytes) that can vary between individuals. Whether these cell types are equally epigenetically affected by environmental exposures is currently unknown, and hence the magnitude of their individual contribution to whole cord blood methylation status may vary. However, in terms of intrauterine exposures and developmental outcomes, whole cord blood DNA methylation levels have been assessed and correlated with various maternal exposures [27,28,29,30,31].

As this is a new and emerging research field, only a limited number of studies are available that have specifically assessed cord blood epigenetic state and matched this with offspring neurodevelopmental outcome. These studies are outlined in Table 1 and reviewed below. We also have included studies which have assessed DNA methylation in other tissues collected at birth or in early life, which has also been linked to offspring neurodevelopment (Table 2). These studies are included to specifically highlight the potential for epigenetic biomarkers to be used as early predictors of neurodevelopment. Lastly, we include studies that assess DNA methylation in the offspring following adverse maternal exposures that are well known to exert detrimental effects on child neurodevelopment. While these latter studies are limited in that they lack long-term follow-up, they are specifically included to build the evidence supporting the potential capacity for DNA methylation to predict child neurodevelopmental outcomes.

## 3. DNA Methylation Biomarkers of Neurodevelopment Outcome

### 3.1. Targeted versus Genome Wide Approach in Practise

Most studies that have assessed epigenetic biomarkers associated with neurodevelopmental outcomes to date have adopted approaches targeting genes in pathways that are known to be susceptible to prenatal programming effects (e.g., stress axis) [25,32] and/or that directly influence brain function throughout life (e.g., neurotrophins, neurotransmitters) [33,34]. These approaches have been useful in understanding the impact of early life exposures on gene regulation and early perinatal outcomes, with only few assessing the longitudinal impact on neurodevelopment (e.g., [35]). Very few studies have adopted whole epigenome analyses to investigate prenatal programming effects, despite their potential to inform novel pathways critical for neurodevelopment. Such insights have been gained from large birth cohort studies, where whole genome methylation analyses of cord blood at birth has been assessed with respect to the child’s later cognitive and behavioral outcomes. For example, in the Southampton Women’s Survey, child IQ at four years was positively associated with cord blood methylation of two CpG loci in *HES1*, a gene that encodes a transcription factor involved in neuronal cell proliferation and differentiation, and in diencephalon development [36]. This association was adjusted for potential confounding by maternal IQ, birth weight and maternal smoking. The positive association between *HES1* methylation and child cognitive development was validated in a further 200 cord blood samples from the Southampton Women’s Survey cohort, where more specific child cognitive functions were assessed at age seven years, including cognitive flexibility, executive function and memory [36]. This association between *HES1* methylation and neurodevelopment extends to child behavioral outcomes. In one-year-old children from the Growing Up in Singapore Towards Healthy Outcomes (GUSTO) cohort, lower externalizing behavior scores were also associated with greater cord blood *HES1* methylation [36]. These studies conducted in large cohorts highlight the potential for genome wide DNA methylation studies to identify biomarkers of early neurodevelopmental outcome (Table 1).

### 3.2. Stress Exposure in Early Life

A large body of evidence exists linking pre- and early post-natal stress exposures with altered fetal and infant brain development. Examples of such stress effects include those induced following acute and chronic maternal stress exposures (e.g., natural disaster, domestic abuse and violence), malnutrition (famine) and exposure to toxins (including alcohol, tobacco, bisphenol A, heavy metals). The leading biological model explaining how these early life exposures result in an increased vulnerability to poor neurodevelopment in the offspring is through excess fetal exposure to stress-induced hormones, such as glucocorticoids (cortisol in humans and guinea pigs, corticosterone in rats and mice). Excess glucocorticoid exposure has widespread effects on the developing brain, including delayed neuronal maturation, myelination, vascularization, and synapse formation [49], and results in cognitive deficits and behavioral difficulties throughout life [49,50,51]. For example, in humans, stress-induced increases in maternal cortisol during pregnancy are associated with lower scores on physical and mental development indices of the Bayley Scales of Infant Development in offspring at three and eight months of age [52], as well as lower scores in adolescence on the Vocabulary and Block design tasks of the Wechsler Intelligence Scale for Children [53], measures indicative of global IQ. Increased prenatal cortisol exposure also impacts on offspring psycho-social behavior, resulting in increased impulsivity and reactive-type behaviors, attention problems, emotional and temperament difficulties and childhood behavioral and conduct problems [54].

Increased cortisol exposure of the developing fetus following maternal stress exposure is facilitated by a decrease in placental cortisol inactivation, through reduced activity of the 11 beta hydroxy-steroid dehydrogenase type 2 (11βHSD2) enzyme. This results in excess cortisol crossing to the fetus, ultimately perturbing fetal growth and development. Decreased 11βHSD2 activity and expression have been observed in pregnancies complicated by various prenatal acute and chronic stress exposures and maternal mental health conditions (such as anxiety and depression) [55,56]. Excess cortisol exposure in utero can also result in lifelong alterations to the offspring’s hypothalamic pituitary adrenal (HPA) axis, the principle neuroendocrine pathway regulating both diurnal and stress-induced cortisol levels. Typically, exposed offspring exhibit decreased basal cortisol levels and increased cortisol responses to stress [49].

Promise for cord blood epigenetic status of genes involved in HPA function or cortisol metabolism to predict poor neurodevelopment has arisen from a number of animal and human studies. In this context, the most extensively phenotyped genes include *NR3C1* which encodes the glucocorticoid receptor and *HSD11B2*, encoding 11βHSD2 [57]. The human glucocorticoid receptor gene (*NR3C1*) consists of 9 exons, with multiple transcripts arising from alternate exon 1 promoter regions, which all translate various isoforms of the same protein. In rats, studies have typically reported the exon 1F promoter region as the most susceptible to altered methylation state following prenatal stress exposure. Human studies are consistent with those from rodents, with increased methylation of the equivalent homolog of the rat *Nr3c1* promoter region observed following early life stress [25,32]. In humans, methylation status of *NR3C1* and *HSD11B2* in the placenta has been assessed for associations with neurodevelopmental indices. In one such study, DNA methylation of the *HSD11B2* gene in placenta from term deliveries was linked with infant’s neuro-behavior assessed prior to hospital discharge [58]. The extent of *HSD11B2* DNA methylation was greater in intrauterine growth restricted infants, and was inversely associated with poorer quality of movements measured on the Neonatal Intensive Care Unit Network Neurobehavioral Scales (NNNS). While this study did not assess longer term neurodevelopmental outcomes, previous studies have shown that poor quality of movements measured on the NNNS is predictive of a lower score on the Bayley’s Psychomotor Developmental Index at 18–24 months of age [59,60] and a greater degree of motor, language and concept problems at age four years [60]. These associations illustrate the potential power of early biomarkers to predict longer term outcomes. While identification of epigenetic modifications has not been limited to the cortisol signaling pathway, it has received the most attention due to the prevailing recognition of excess cortisol as a common convergent pathway in developmental programming events.

To investigate the direct consequences of excess prenatal glucocorticoid exposure on the epigenetic landscape of brain tissue, Matthews and colleagues exposed pregnant guinea pigs to the synthetic glucocorticoid betamethasone during periods of rapid neurogenesis and brain growth (Days 41–42 and 51–52 of pregnancy, respectively) [61]. DNA and RNA were extracted from offspring hippocampal tissue either on post-natal Day 52 or 65, and microarray analysis with *Nr3c1* DNA immunoprecipitation and chip hybridization performed for those genes that demonstrated differential *Nr3c1* DNA binding compared to control (unexposed) offspring. While more than 1000 genes were differentially expressed between control and betamethasone-exposed offspring on Day 52, only 81 genes remained differentially expressed between the two groups by Day 65. Exposure to the synthetic glucocorticoid therefore appeared to result in precocious development of the guinea pig hippocampus. However, *Nr3c1* DNA binding and DNA methylation were differentially affected by the betamethasone treatment. Specifically, hypomethylation was observed in promoter regions of genes encoding *Eme2* and *Cntrob*, while hypermethylation of *Gnpda1* was observed in hippocampi of betamethasone compared to control offspring [61]. These findings suggest activation of specific pathways in brain development following prenatal glucocorticoid exposure. Whether these directly contribute to the altered stress responses and behavioral changes exhibited by offspring following glucocorticoid exposure in utero are yet to be determined. Nonetheless, these results clearly indicate that prenatal exposure to glucocorticoids results in differential DNA methylation in the hippocampus, illustrating the potential for this to be used as a biomarker for offspring neurodevelopmental outcome.

### 3.3. Prenatal Exposure to Natural Disasters

In humans, longitudinal studies of women and children following exposure to natural disasters in pregnancy have allowed the relationship between both prenatal objective and subjective stress on child developmental outcomes and DNA methylation to be determined. Such studies have been performed in pregnant women exposed to the 1998 Quebec Ice Storm, where both prenatal stress could be measured objectively (quantified by period of hardship experienced, with the power outage lasting up to six weeks) and subjectively (through women’s perceived stress rating of this period). This dual assessment has allowed for the impacts of stress on child development and DNA methylation status to be determined. In this cohort, higher maternal subjective stress ratings were associated with subsequent infant temperament [62], child behavior (aggression) and mental health measures (depression and anxiety) [63], while objective stress measures (period of time without power) were associated with cognitive performance (IQ and language scores) in toddlers (age two years) [64] and children (age five years) [65]. At age eight and 13 years, peripheral blood and saliva were collected to assess DNA methylation using a genome wide approach. Objective stress was correlated with DNA methylation of over 1600 CpGs in a dose–response relationship [66]. Nine of these genes containing 12 of the top 500 CpGs identified were validated, and found to be predominantly involved in immune function. Further, correlations found between DNA methylation in T cells, peripheral blood mononuclear cells and DNA extracted from saliva, indicate a system-wide DNA methylation response to this prenatal stress exposure [66]. Although the DNA methylation status of these genes was determined in childhood, these studies further support the existence of an epigenetic signature that could be used to determine future risk of poor neurodevelopmental outcome.

### 3.4. Maternal Depression and Anxiety in Pregnancy

Maternal anxiety and depression during pregnancy are associated with poor child behavioral and cognitive outcomes, and an increased incidence of child mental health problems (reviewed in [67]). Data from the Avon Longitudinal Study of Parents and Children (ALSPAC) cohort, indicate that high levels of maternal anxiety, specifically during pregnancy, doubled the risk of the 4–7-year-old children having emotional and behavioral problems [68]. These neurobehavioral disorders in childhood may be identified by altered epigenetic processes induced by different maternal mental health states. Early work in rodents exploring this relationship identified that reduced maternal licking and grooming behavior in the first few days of life resulted in increased DNA methylation of the exon 1–7 promoter of the *Nr3c1* gene in the rat hippocampus, accompanied by decreased *Nr3c1* expression [25] (Table 2). These early life changes persist into adulthood, and are associated with increased anxiety like behaviors [25]. Similar changes in cord blood *NR3C1* methylation have been reported in human neonates that correspond to maternal mental well-being. In an Australian birth cohort (Barwon Infant Study), maternal depression and anxiety at 28 weeks’ gestation were nominally associated with increased DNA methylation of different sites in the *NR3C1* 1F promoter region in cord blood mononuclear cells [37]. While these associations did not withstand statistical corrections for multiple comparisons, other studies directly support these findings. For example, maternal depression in the third trimester of pregnancy has also been associated with altered methylation of *NR3C1* gene promoter regions in cord blood mononuclear cells, and this corresponded to altered stress responses in the three month old infants [35]. Altered DNA methylation of other gene regions has also been reported with maternal anxiety, including changes in the imprinting control region of *IGF2/H19* [38]. Additional analyses of these data showed that when segregated by birth weight, the relationship between DNA methylation and maternal anxiety was stronger in the lower birthweight group (≤3530 g) and when stratified by sex, the relationship was only seen in females [38]. Together, studies in humans and rats demonstrate that maternal mental health and behavior towards her offspring in very early life can result in epigenetic modifications, which appear to increase vulnerability to poor long term neurodevelopmental outcomes.

The potential reversibility of epigenetic changes following maternal neglect and depression has been investigated. In rats, the consequences of maternal neglect in the early life environment on *NR3C1* methylation, HPA reactivity and anxiety behaviors can be reversed by increased tactile stimulation in the postnatal environment [25,69,70]. In humans, maternal stroking of their infant in early life following pre- and post-natal depression appears to have similar beneficial effects. This has been explored in mothers who participated in the Wirral Child Health and Development Study (UK) [71]. Both pre- and post-natal depression and anxiety were assessed, as well as maternal stroking at five and nine weeks of age, using the self-report Parent-Infant Caregiving Scale. Infants were followed postnatally, to assess infant emotionality using the Distress to Limitations Scale and the Infant Behavioral Questionnaire [72], as well as the Child Behavior Checklist at 2.5 years [73]. A sub-set of mothers and infants also provided saliva samples for targeted DNA methylation analysis of the *NR3C1* gene at age 14 months. High prenatal depression and anxiety scores, together with low stroking behavior in early life, were associated with worse infant negative emotionality [72] (Table 2), and child internalizing behaviors and anxiety and depression symptoms at age 2.5 years [73]. This relationship was not observed in infants of greater stroking mothers. In this study, it was difficult to separate the individual impact of prenatal from postnatal depression, given that most women who experience prenatal depression also have postnatal depression. It was, however, possible to identify the impact of postnatal depression in the absence of prenatal depression. At 14 months, the percentage of *NR3C1* methylation in saliva from the infant was found to increase with worsening postnatal depression, but only in infants of mothers who reported low prenatal depression. There was no change in DNA methylation patterns in infants of mothers who reported both high prenatal and postnatal depression scores. While the number of DNA samples from infants of women who had low prenatal but high postnatal depression was low, infants of these mothers had higher DNA methylation levels than all other infants. Interestingly, if the mothers in this group reported high stroking behavior at five weeks of age, DNA methylation levels in infant saliva fell to those observed in the other children. This effect was not observed at nine weeks of age, indicating a potential critical period for reversing infant methylation changes induced by maternal depression. The results of these studies are promising, indicating that the effects of somatosensory deprivation on the child during early periods of development, commonly occurring with postnatal depression, may be reversible with targeted interventions.

The consequences of pre- and post-natal depression on the methylation status of the *NR3C1* and *HSD11B2* genes have also been assessed in the placenta. In a study of 482 mother–term-born infant pairs (13.7% of whom had depression and/or anxiety) [74], maternal depression was associated with increased methylation of the *NR3C1* CpG2 site (Table 2). This hypermethylation state in the placenta of mothers with depression was associated with infant behavioral indices (greater lethargy and hypotonia), as well as neonatal self-regulation scores. Maternal anxiety had no impact on *NR3C1* CpG2 methylation state, yet was associated with increased methylation of the placental *HSD11B2* gene. Greater methylation with maternal anxiety was associated with increased infant hypotonia. Given that the placenta regulates fetal glucocorticoid exposure, greater DNA methylation of these genes may confer increased cortisol exposure in utero. This may be a mechanism contributing to the poor neurodevelopmental outcomes observed with poor maternal mental health states.

While poor infant outcomes following maternal depression have been documented in the literature, controversy exists over whether pharmacological treatment of maternal depression is beneficial. Guidelines from the USA, UK and Australia all recognize the limited or inadequate evidence around the safe use of anti-depressants in pregnancy, given that risk factors for poor pregnancy and child health outcomes, including smoking, obesity and preterm birth, occur more frequently in women with depression compared to healthy women [79,80,81]. It is not surprising, then, that conflicting reports of unique epigenetic modifications have been found with medicated versus non-medicated depression in pregnancy. Compared to healthy controls, cord blood from neonates of mothers with non-medicated depression demonstrated 42 CpG sites with altered DNA methylation [82]. However, when compared to infants of mothers who took anti-depressant medication through pregnancy, no changes in genome-wide DNA methylation were observed [82]. Conversely, another study reported no difference in genome-wide DNA methylation in cord blood between infants of medicated and non-medicated women with depression and controls [83], highlighting the complexity of this condition.

Sex of the fetus may also play an important mediating role in determining DNA *NR3C1* methylation response to maternal depression. Male infants exposed to maternal depression in second or third trimester showed increased *NR3C1* DNA methylation in buccal swabs, while female infants demonstrated hypomethylation in response to the same exposure [33] (Table 2). Few studies have examined sex-specific effects on DNA methylation, despite repeated reports of sex-dependent outcomes in male and female offspring following maternal stress exposure in both animal and human research. This work suggests that sex-specific changes in DNA methylation following prenatal exposures may be a contributing mechanism underlying the sex-specific effects of prenatal stress reported in the literature [33]. 

Other genes that appear to be susceptible to changes in methylation with prenatal depression include those encoding neurotrophins, such as brain derived neurotrophic factor (*BDNF*) [84]. *BDNF* is a neuronal growth factor with multiple roles in neuronal proliferation, differentiation, growth, apoptosis and synaptic plasticity across development and the lifespan. In a prospective study of mother—infant dyads, maternal depression was assessed during either second or third trimester, using the Edinburgh Post-natal Depression Scale, and buccal swabs were collected from infants at age two months. Using a targeted approach to assess methylation at five CpG sites within the *BDNF* promoter IV region, decreased DNA methylation was observed at CpG3 following prenatal depression [33]. The consequence of this epigenetic change is yet to be determined, but altered DNA methylation of this region has been observed in animal models following adverse prenatal exposures [31], potentially indicating a gene highly susceptible to epigenetic alterations following early life adversity. Given that *BDNF* is involved in many developmental processes in the fetal brain, as well as ongoing synaptic function and plasticity, altered DNA methylation of this gene may have quite detrimental consequences for neurodevelopment. 

The epigenetic impact of maternal depression in pregnancy has also been investigated in pathways regulating neurotransmitter uptake, in particular serotonin (5-HT). Serotonin is constitutively expressed in neurons, with its uptake regulated by the serotonin transporter (5-HTT). While best known as a neurotransmitter, serotonin also has critical neurotrophic roles during development, mediating neuronal growth and differentiation [85]. Variations in 5-HTT expression and serotonin reuptake efficiency have been attributed to a variant in the promoter region of the gene that encodes 5-HTT (*SLC6A4*) and DNA methylation of the *SLC6A4* promoter region [86]. Maternal depression during the second trimester has been associated with decreased methylation of the *SLC6A4* promoter region (at CpG sites 6 and 9) in cord blood [34]. This association was not altered by use of anti-depressant medication nor by sex of the infant. These results suggest that maternal mood (depression) can alter DNA methylation of the *SLC6A4* gene promoter, and therefore may contribute to altered *SLC6A4* expression in the offspring. Decreased expression of *SLC6A4* during development may have long term consequences on child neurodevelopment, via increasing 5-HT reuptake. The consequences of altered 5-HT reuptake may include physical, emotional and/or behavioral disturbances throughout life, given that serotonin plays key functions in regulating mood, sleep and cognitive performance. Future longitudinal studies of these children will increase our understanding of the longer-term impact of maternal depression and child *SLC6A4* methylation on neurodevelopment.

### 3.5. Exposure to Maltreatment in Early Childhood

Maltreatment in childhood, including physical, sexual and emotional abuse or neglect, can have lasting effects on a child’s mental health, increasing the risk of multiple psychopathologies, such as depression, anxiety and borderline personality disorder, and increasing the risk for suicide [87]. The contribution of epigenetic alterations to these poor mental health outcomes is increasingly being recognized, and given that altered HPA signaling is a common feature of many psychopathologies, particular attention has once again been directed towards studying epigenetic modifications of genes involved in this axis. For example, post-mortem analyses of brain tissue from suicide victims have identified increased *NR3C1* DNA methylation in those exposed to child maltreatment [88,89]. Similarly, increased DNA methylation of *NR3C1* is observed in blood of patients with borderline personality disorder (BPD) [90]. This association between early life maltreatment, BPD and altered *NR3C1* methylation has been explored more recently in 11–21 year olds. Participants completed a comprehensive suite of psychological health assessments, evaluating behavioral, perceived and standardized symptomology, as well as providing peripheral blood for DNA methylation in lymphocytes to be determined at 41 CpG sites spanning the *NR3C1* gene [75] (Table 2). A strong relationship was demonstrated between increased DNA methylation of the *NR3C1* exon 1F promoter region and childhood maltreatment and poor psychological health. Specifically, early life childhood maltreatment was associated with increased methylation of a CpG site located in the exon 1F promoter region, which was highly correlated with the development and severity of borderline personality symptoms. Methylation of a second CpG site located in the *NR3C1* exon 1E promoter region was also associated with depression symptoms, with methylation at both sites inversely associated with health related quality of life. 

Genome wide approaches have also been used to investigate the long term impact of childhood abuse on the epigenome. Adult men (aged 45 years) who took part in the 1958 British Birth Cohort study reported the degree of childhood abuse experienced in the first 16 years of life, and provided peripheral blood for DNA analysis [76]. Over 900 gene promoter regions were found to be differentially expressed between those who reported high levels of abuse compared to those who reported none. These genes were found to be enriched in pathways involving regulatory and developmental functions. The association of child abuse with altered DNA methylation in peripheral blood at age 45 suggests a system wide adjustment to this early life exposure, which persists into adulthood. While causality cannot be determined, this system wide approach to detect altered DNA methylation of multiple gene regions provides further evidence that epigenetic modifications can be linked to early life adverse exposures, with the potential for genome wide approaches to be used to identify a unique epiphenotype that predicts later poor health outcome. 

Quality of parental care provision in early life can also determine child and adult mental health outcomes. Low maternal care increases the risk of poor mental health in later life [77]. The biological mechanism contributing to these outcomes are not well-defined but altered DNA methylation following maternal deprivation during early life has been observed and has been associated with changes in offspring mental health and behavioral responses to stress. Studies of infant rhesus macaques that were either maternally raised or removed from their mother on Post-Natal Day 1 and then nursery reared, have shed light on how early life parental interactions influence DNA methylation. Compared to infants raised by their mother, maternally deprived infants exhibited higher DNA methylation of the *SLC6A4* gene in peripheral blood mononuclear cells, and demonstrated the greatest activity during a period of enforced social isolation [91]. In those infants who were maternally reared, lower maternal care behaviors towards her infant in early life were associated with a higher degree of *SLC6A4* methylation, indicating a gradient response. In humans, poor maternal care provision has also been associated with altered DNA methylation. Increased methylation of the *BDNF* and oxytocin receptor genes in whole blood has been observed in adults who report low maternal care provision during the first 16 years of life [78]. Notably, changes in DNA methylation were only observed in some regions of these genes but not others, indicating a specific DNA methylation response to this exposure. Together, these human and animal data show that early maternal care is a critical determinant of child behavioral responses, and strongly support altered DNA methylation as a reflection of these outcomes (Table 2). These epigenetic changes may contribute to a heightened risk of poor mental health in later life and increased stress reactivity [92]. This study also highlights that different regions of the same gene are differentially affected by the early life environment. Such results highlight the potential to identify an epigenetic signature that can predict child health outcome with high sensitivity and specificity. More research in this area will inform this epiphenotype.

### 3.6. Exposure to Toxic Heavy Metals 

Numerous heavy metals such as mercury, cadmium and arsenic are known to impact fetal development, and prenatal exposures of these heavy metals are associated with poor child outcomes [93]. The World Health Organization lists mercury, cadmium and arsenic as part of the 10 chemicals that are of a major public health concern. Prenatal exposures to toxins such as heavy metals can alter DNA methylation profiles and in fact can be used as potential biomarkers for exposures [94]. Recently there have been several studies that have examined prenatal exposure to heavy metals and DNA methylation in cord blood (Table 1). There have been two studies that have assessed maternal mercury levels during pregnancy and DNA methylation changes in cord blood. One of these used a genome wide approach by assessing DNA methylation in 138 mother–infant pairs by Illumina 450K Methylation Array but found no changes in cord blood DNA methylation associated with maternal plasma mercury or arsenic levels after Bonferroni correction [39]. However, Bakulski and colleagues, in a similar number of mother–infant pairs assessed a smaller number of genes and found cord blood DNA methylation changes in *ANGPT2* and *PRPF18* were associated with first trimester maternal circulating mercury levels [40]. Given that increased maternal mercury exposure is associated with poor child neurodevelopment, these findings highlight the potential for epigenetic markers in cord blood to be used to identify children at risk, in the absence of information about maternal environmental exposures experienced during pregnancy (Table 1). However, the absence of similar findings between studies warrants caution with interpretation, with further studies being required to understand the epigenetic impact of these exposures.

The impact of cadmium on cord blood methylation state has also been investigated. Out of the three studies that have assessed cadmium, two found maternal circulating cadmium levels were significantly associated with cord blood DNA methylation changes. The largest of these studies included 319 infant–mother pairs, assessed nine imprinted genes and showed a significant association between maternal cadmium concentrations and altered cord blood DNA methylation at the *PEG3* differentially methylated region (DMR) [41]. A smaller study of 17 mother–baby pairs assessed over 4.5 million CpG sites and found cord blood DNA methylation of 61 genes was significantly associated with maternal cadmium levels [42]. In the final study, assessing 127 mother child pairs, an Illumina 450K DNA Methylation Array was used and no significant associations between maternal blood cadmium levels and cord blood DNA methylation levels were identified [43]. These contrasting findings are difficult to interpret, particularly given the limited number of matched samples from mother and infant pairs and the different approaches used to assess DNA methylation (Table 1). Taken together, these studies indicate that maternal cadmium exposure may exert epigenetic changes on the developing infant, and could therefore potentially contribute to the poor neurodevelopmental outcomes seen with such exposure. However, further study is needed to clarify these preliminary findings.

Several other studies have investigated whether maternal arsenic levels are associated with DNA methylation changes in cord blood. In the same cohort mentioned above in which mercury was investigated, there were no changes in cord blood DNA methylation associated with maternal arsenic levels [39]. However, this is not consistent with other studies. In a cohort with higher levels and greater range of arsenic exposure, cord blood DNA methylation levels in 4771 probes (2919 genes) were significantly associated with maternal arsenic levels [44]. This was supported by investigations of maternal urinary arsenic levels in early and late pregnancy in 127 mother–infant pairs, which found an association between these and cord blood DNA methylation changes [45]. Interestingly, these changes were more evident in boys than girls and stronger associations were seen with early compared to late pregnancy arsenic levels [45]. This study also utilized the Illumina 450K Methylation Array and, after adjusting for multiple comparisons, only three CpG sites in boys and none in girls remained significantly correlated with arsenic levels [45]. Kile and colleagues assessed the relationship between arsenic contamination of maternal drinking water in first trimester and DNA methylation in cord blood in 44 mother–baby pairs using Illumina 450K Methylation arrays [46]. They reported that prenatal exposure to arsenic altered T cell subpopulations in cord blood (increasing the proportion of CD8+ T cells and decreasing the proportion of CD4+ T cells), but also altered DNA methylation in cord blood, even after adjusting for altered leukocyte distribution [46]. Although, several of the studies above have shown that maternal arsenic levels during pregnancy are associated with DNA methylation changes in cord blood, there is an equal number of studies with similar population sizes that have shown no correlations [47,48]. However, limitations in comparing these studies include the method for measurement of arsenic levels, the timing of the measurements and the variability and range of arsenic exposures in these different cohorts (Table 1). As with the mercury studies, larger cohorts are required before conclusively being able to determine if exposure to these heavy metals during pregnancy impacts cord blood DNA methylation. In addition, longer term studies are needed to determine if the heavy metal exposures impact neurodevelopmental outcome in the children and if this can be predicted by DNA methylation changes in cord blood. Recent studies have shown that arsenic exposure is also associated with DNA methylation changes in the placenta [30,95]. Whether these changes correspond to neurodevelopmental outcome requires further investigation.

## 4. Conclusions

Epigenetic changes that occur in response to an adverse prenatal environment may constitute a biological mechanism by which early adversity results in long term changes to child health, contributing, or increasing susceptibility, to pathology. Existing data support differences in DNA methylation of specific genes (e.g., the glucocorticoid receptor, serotonin transporter and *BDNF*) following exposures to prenatal and early life stress and adversity, as potential factors that may contribute to the postnatal phenotype. While postnatal factors can impact child neurodevelopment, the overarching goal of epigenetic biomarker research is to identify an epigenetic signature that: (i) can discriminate children at risk for poor neurodevelopmental health outcomes as early as possible, so that limited health funding resources can be directed to those most at risk; and (ii) identify novel mechanistic pathways that can inform new interventions. Early identification tools and interventions designed to address cognitive and mental health disparities, particularly in low socio-economic areas, are urgently required to alleviate the associated health, social and economic burden. To achieve this, what is needed is an unbiased approach to assess DNA methylation in a genome wide manner without the bias of “candidate genes” or “brain specific genes”. The identification of biomarkers that can predict neurodevelopmental outcome does not require that the genes have some brain related function, despite these genes receiving the most research attention to date. In fact, DNA methylation of CpG sites or regions that are not associated with genes, will still serve as great biomarkers provided they can reliably predict neurodevelopmental outcomes. What is now needed are genome wide DNA methylation approaches in cord blood that can identify children at risk who will require early interventions. These studies will need to take into account different cell compositions and the potential impact this has on DNA methylation profiles. Further, epigenome wide analyses that are unbiased would be most beneficial to increasing our understanding in this area, and should be performed in multiple large cohorts.

## Figures and Tables

**Table 1:** Summary of studies assessing changes in cord blood DNA methylation and their association with an adverse prenatal exposure and/or child neurodevelopmental outcome.

Reference	Sample Size	Method	Gene/s Exhibiting Difference in DNA Methylation	Adverse Early Life Exposure	Child Neurodevelopmental Outcome
[34]	82	Targeted	*SLC6A4*	Maternal depression (second trimester)	-
[35]	82	Targeted	*NR3C1*	Prenatal depression and anxiety	Altered stress response (age 3 months)
[36]	175	Promoters of 25,000 genes (*n* = 24), Targeted (*n* = 175)	*HES1*	-	IQ (age 4 years)
[36]	200	Targeted	*HES1*	-	Cognitive flexibility, executive function, memory (age 7 years)
[36]	108	Targeted	*HES1*	-	Externalizing behavior (age 1 year)
[37]	481	Targeted	*NR3C1*	Maternal depression and anxiety	-
[38]	576	Targeted	*IGF2* and *H19*	Maternal anxiety	-
[39]	138	Illumina Infinium Methylation450K array	No changes	Mercury exposure	-
			No changes	Arsenic exposures	-
[40]	85	Targeted	*ANGPT2* and *PRPF18*	Mercury exposure	-
[41]	319	Targeted	*PEG3*	Cadmium exposure	-
[42]	17	Methylated CpG island recovery assay	61 genes	Cadmium exposure	-
[43]	127	Illumina Infinium Methylation450K array	No changes	Cadmium exposure	-
[44]	38	Illumina Infinium Methylation450K array	2919 genes	Arsenic exposure	-
[45]	127	Illumina Infinium Methylation450K array	3 CpG sites associated with arsenic (males only)	Arsenic exposure	-
[46]	44	Illumina Infinium Methylation450K array	Genome wide DNA methylation levels associated with arsenic exposure	Arsenic exposure	-
[47]	134	Illumina Infinium Methylation450K array	No changes	Arsenic exposure	-
[48]	101	[3H]-methyl-incorporation assay, Alu, LINE-1 and LUMA.	Non-significant changes	Arsenic exposure	-

**Table 2:** Summary of studies assessing changes in tissue DNA methylation associated with an adverse prenatal exposure and/or child neurodevelopmental outcome.

Reference	Species	Method	Tissue	Gene/s Exhibiting Difference in DNA Methylation	Adverse Early life Exposure	Child Outcome
[25]	Rat	Targeted	Hippocampus	*Nr3c1*	Low maternal grooming	Increased anxiety like behaviors
[33]	Human *n* = 57	Targeted	Buccal swabs (at age 2 months)	*BDNF*	Maternal depression	-
[58]	Human *n* = 185	Targeted	Placenta	*HSD11B2*	-	Poor quality of movement (age < 4 days)
[66]	Human *n* = 36	Illumina Infinium Methylation450K array	Saliva (age 8 years)	*SCG5* and *LTA*	Maternal stress	-
			Peripheral blood T cells (age 13 years)	Over 1600 CpG sites		
[72]	Human *n* = 181	Targeted	Saliva (age 14 months)	*NR3C1*	Postnatal depression in absence of prenatal depression	-
[74]	Human *n* = 482	Targeted	Placenta	*NR3C1*	Prenatal depression	Increased lethargy, increased hypotonia, decreased self-regulation (age < 4 days)
				*HSD11B2*	Maternal anxiety	Increased hypotonia (age < 4 days)
[75]	Human *n* = 46	Targeted	Peripheral blood (age 11–21 years)	*NR3C1*	Childhood maltreatment	Borderline personality symptoms
[76]	Human *n* = 40	Methylated DNA immunoprecipitation	Peripheral blood (men, 45 years)	>900 gene promoter regions	Childhood abuse (first 16 years of life)	Depression symptoms
[77]	Rhesus macaques	Targeted	Peripheral blood mononuclear cells	*SLC6A4*	Maternal deprivation	Increased activity during social isolation
[78]	Human *n* = 85	Targeted	Whole blood (adults)	*BDNF* and oxytocin receptor	Poor maternal care first 16 years	-

## Abbreviations

The following abbreviations are used in this manuscript:

APIB	Assessment of Preterm Infants Behavior
BPD	borderline personality disorder
HPA	hypothalamic pituitary adrenal
MRI	magnetic resonance imaging
NNNS	Neonatal Intensive Care Network Neurobehavioral Scale
